# Reclamation of Cultivated Land Reserves in Northeast China: Indigenous Ecological Insecurity Underlying National Food Security

**DOI:** 10.3390/ijerph17041211

**Published:** 2020-02-13

**Authors:** Wenbo Li, Dongyan Wang, Shuhan Liu, Yuanli Zhu, Zhuoran Yan

**Affiliations:** 1 College of Earth Sciences, Jilin University, Changchun 130061, China; wb_li@jlu.edu.cn (W.L.); zhuyl16@mails.jlu.edu.cn (Y.Z.); yanzr19@mails.jlu.edu.cn (Z.Y.); 2 Institute of Land Management, Northeastern University, Shenyang 110169, China; liushuhan@wfxy.neu.edu.cn

**Keywords:** land reclamation, requisition–compensation balance policy, ecological security, food security, ecosystem services, rapid urbanization

## Abstract

The competition for land resources created by the need for food security and ecological security is intensifying globally. To resolve the issue of land scarcity in agriculture following rapid urbanization, China implemented its requisition–compensation balance policy of cultivated lands in 1997, the introduction of which consumed numerous areas of land, such as river shoal and bare land, through reclamation. Moreover, these reclaimed and newly cultivated lands were mainly distributed in the northern part of China. Most previous studies of this subject have only examined the overall balance of cultivated lands in well-developed regions, and there is a lack of knowledge about the indigenous gains and losses before and after reclamation in important areas such as northeast China. Therefore, this study selected two representative county-level units in northeast China as the study area to analyze the conversion of cultivated land reserves during 1996–2015, evaluate the performance of reclaimed cultivated lands in terms of quality and productivity and calculate reclamation-induced changes in ecosystem service value. The results indicated that by 2015 only 16.02% of the original cultivated land reserves remained unconverted; nearly 60% were reclaimed as cultivated lands and over 20% were converted to other land resources. River shoal and ruderal land were the primary resources for cultivated lands compensation, and marsh, bare land and saline-alkaline land were found to be converted the most thoroughly. The gain of 23018.55 ha reclaimed cultivated lands were of relatively inferior quality and lower productivity, contributing approximately 4.32% of total grain output. However, this modest gain was at the expense of a 768.03 million yuan ecosystem services loss, with regulating services and supporting services being undermined the most. We argue that even if northeast China continues to shoulder the responsibility of compensating for a majority of cultivated land losses, it still needs to carefully process reclamation and introduce practical measures to protect indigenous ecosystems, in order to better serve the local residents and ensure prolonged food security with sustainability.

## 1. Introduction

In a world pervaded with rapid urbanization and booming populations, food security has always been a primary challenge [1]. Sustaining enough grain yield, promotion of modern agricultural techniques and sufficient cultivated land resources with guaranteed quality are essential [2,3]. However, cultivated land resources worldwide are now confronted with strong pressure of being occupied by not merely urban but also rural expansion [4,5,6]. With the purpose of compensating for this loss, reclamation of natural resources such as grasslands is often seen as a convenient and accessible pathway, one that will resolve the issue of land scarcity for agriculture in the short run [7,8]. However, this compensation certainly comes with serious ecological consequences, such as deforestation [7,9], biodiversity decline [10], degradation of water quality [11] and habitat fragmentation [12]. How to make prudent trade-off decisions between food security and ecological security, in this regard, becomes a vital issue that needs to be addressed promptly for achieving and coordinating between the Sustainable Development Goals (SDGs) [13,14].

China has implemented the requisition–compensation balance policy since 1997 to control the gross of cultivated land through reclamation of other land resources, denoted as ‘the cultivated land reserves’ [15,16]. Globally, the policy could be perceived as a representative large-scale reclamation strategy associated with rapid urbanization and agricultural expansion. In view of the potential ecological consequences, it has long since been suggested by scholars that the environmental effects of such a strategy must be evaluated [17]. Indeed, there are in-depth studies that have compared the occupied and reclaimed cultivated lands in terms of quantity, quality and ecosystem services, proving that the dynamic balance purpose was not realized [8,18]. However, few efforts have been made to weigh the indigenous gains and losses before and after reclamation. Furthermore, because the policy allows the balancing to be carried out across regions, the regions with abundant cultivated land reserves (such as northeast and northwest China) are actually taking on more ecological risk resulting from the reclamation [8,19]. Despite this, most previous studies have only focused on the dynamic balance of cultivated lands in well-developed regions, which are dominated by occupation rather than reclamation [18,19,20].

Even if cultivated land is considered as the bottommost of the resources on which humans survive, it fails to contribute to crucial ecosystem services that could only be provisioned by natural resources [21,22]. What is more, an agroecosystem without supports from natural resources or appropriate ecological preservation will be acknowledged as unsustainable [20,23]. Therefore, in order to ensure both food and ecological security, the gains and losses through reclamation in the regions characterized by massive loss of cultivated land reserves must be weighed. Hence, we selected Dehui City and Jiutai District, two neighboring county-level units in northeast China that used to be endowed with abundant cultivated land reserves, to assess gains and losses through reclamation from 1996 to 2015. The objectives of the present study are: (a) to illustrate the conversion of cultivated land reserves, (b) to evaluate the performance of lands reclaimed from different reserves in terms of quality and productivity, and (c) to reveal changes of ecosystem service value (ESV) in reclaimed areas. The results could provide a basis for resolving land use conflicts between agroecosystem and other ecosystems in the major areas of reclamation worldwide.

### 1.1. Progress of Requisition–Compensation Balance Policy in China

China’s per capita cultivated land is far below the world average, and the land resources that could be reclaimed as cultivated lands are scarce due to the country’s complex landforms [1,15]. This suggests that the conservation of cultivated land resources is a top priority. In 1997, for the first time, China explicitly determined to maintain the dynamic balance of existing cultivated lands, mostly in an effort to protect it from reckless and illegal urban occupation [16]. The following year, the requisition–compensation balance policy of cultivated lands was enacted in the revised version of the Land Administration Law [18]. Nevertheless, statistics since 1997 increasingly indicated that the cultivated lands being occupied were mostly flat, concentrated and fertile, while the lands newly cultivated as compensation were remote and suffered from worse farming conditions [15,16]. Alerted to the decline of cultivated land quality, a request for quality balance was supplemented since 2004 with the help of another national project to grade cultivated land [16,24]. Moreover, due to the uneven distribution of cultivated land reserves in China, later on the policy started to allow the compensation project with permission from the State Council to be carried out in separate provinces different from the actual locations of the occupations [18]. Years after the implementation of this policy, however, it was evidenced by relevant quantitative studies that the quality balance was not even close to being achieved, and both the quantity and quality of cultivated land were found to be more unevenly distributed on a national scale [4,8].

Recently, scholars have suggested that along with the structural reform of ecological civilization in China, ecological balance should also be considered with regard to compensating cultivated lands [9]. Meanwhile, a telecoupling analysis of the requisition–compensation balance policy indicated that the ecosystem services in the middle reaches of Yangtze River urban agglomerations were on the decline, implying that the ecological balance had not been achieved in well-developed areas of China, at least not for the moment [18]. The quality and ecological imbalance underlying the quantity balance of cultivated land suggests that practical measures should be adopted for building sustainable agriculture, and to develop such measures we should be aware of what, where and how the indigenous land system was influenced by the requisition–compensation balance policy.

### 1.2. Reclamation of Cultivated Land Reserves in Northeast China

By way of a definition, ‘cultivated land reserves’ are here seen as non-agricultural land resources, such as mining land and bare land, that have the potential to be reclaimed as cultivated land under certain conditions [25]. China is a large country covered with many mountainous and deserted areas, and cultivated land reserves are scarce [1,16]. According to the national survey and the assessment of cultivated land reserves (an assessment that illustrates the spatial suitability for reclamation), which were initiated in 2000 and 2014, respectively, the cultivated land reserves that could be reclaimed instantly were even more scarce and being increasingly reduced [25]. Furthermore, the available statistics indicated that most of the cultivated land reserves were distributed in the northern part of China; the north-west part of China was also regarded as an ecologically fragile area where conversion of natural resources was not recommended [8,26].

**Figure 1:**
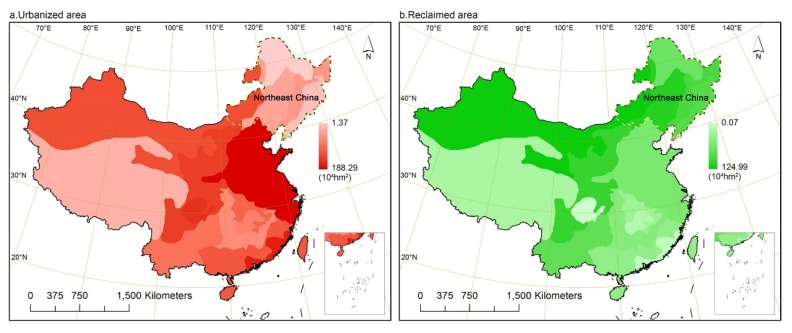
(**a**,**b**) Spatial regionalization and distribution of urbanized areas and reclaimed areas during 2000–2010 in China (the data were mapped from a previous study by Liu [8]).

Previous studies demonstrated that over 95% of the cultivated land reserves in northeast China are reclaimable, and so it became a major area where most reclaimed cultivated lands were concentrated [27]. With reference to a study on spatiotemporal land use changes in China [8], it could be observed that ever since the implementation of the requisition–compensation balance policy most of the built-up land converted from other land resources was concentrated in the eastern coastal area (Figure 1a), while most of the reclaimed cultivated lands were evidently distributed in the northern part (Figure 1b). As a consequence, northeast China could be considered as an area characterized by little occupation but massive compensation activity. When confronted with this noteworthy agricultural expansion, northeast China, serving as one of the country’s most crucial grain bases, needs to reconsider the position of local ecological security and direct it towards the development of sustainable agriculture [23,28]. Unlike the supply of food and raw materials on a worldwide scale, many ecosystem services, such as water conservation and soil retention, are more localized and crucial for indigenous agroecosystems [22,29].

## 2. Materials and Methods

### 2.1. The Study Area

**Figure 2:**
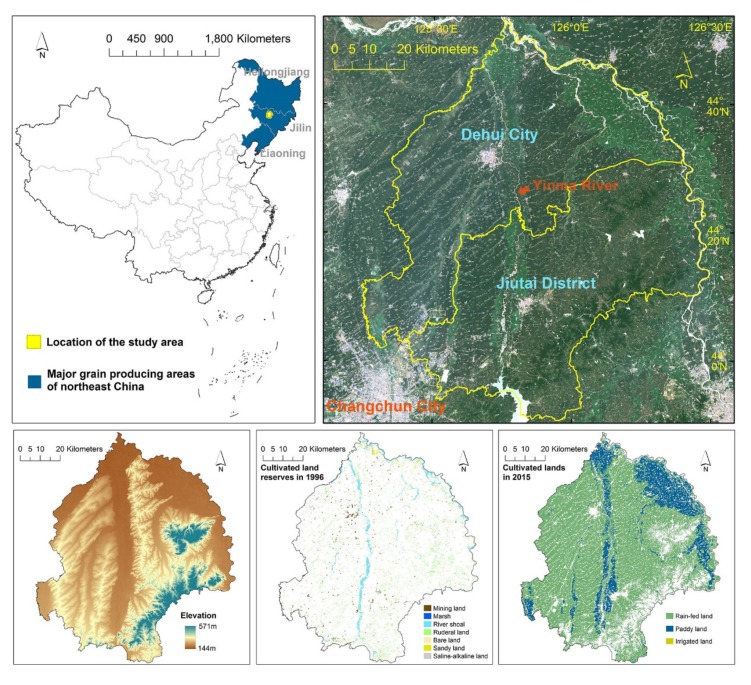
Location of the study area.

Dehui City and Jiutai District are two neighboring county-level units in Jilin Province (Figure 2); they are located in the interior region of Songnen Plain, a region covered with organic matter-rich black soils. Moreover, due to the superior agricultural conditions in this region, they have long been in the top 10% county-level units in the national grain yield rankings. Dehui City and Jiutai District were selected as the study area because: (a) the region was dominated by a plain area and low hilly area (Figure 2) which are the two most widely distributed agricultural landscapes in northeast China; and (b) it was covered with abundant cultivated land reserves in 1996, but most of these resources had been reclaimed as cultivated lands since the requisition–compensation balance policy was implemented (Figure 2).

### 2.2. Data Processing

To begin with, and based on the standard of the aforementioned national survey and the assessment of cultivated land reserves, seven land-use categories-mining land, marsh, river shoal, ruderal land, bare land, sandy land and saline-alkaline land-were selected as the representative cultivated land reserves in the present study. In addition, the cultivated land was further classified as rain-fed land, paddy land and irrigated land. The land use data were derived from the first national land survey database established in 1996 and the detailed land use change survey database in 2015. All land use data were readily obtainable, precise to a parcel level and fully conformed to the requirements of this study. Distribution of cultivated land reserves in 1996 and cultivated lands in 2015 are presented in Figure 2; Table S1 showed detailed connotations for major land use categories in the present study, which were transcribed from the national standard for classifying land use.

### 2.3. Quality Assessment

In order to assess the quality of reclaimed cultivated lands, we selected five quality indexes: soil pH, soil organic matter, top-soil texture, soil depth and drainage condition. The data were obtained from the 2015 quality grading database of cultivated land. Moreover, in consideration of the current situation in the study area, we adjusted the assessment method on the basis of the quality grading method of cultivated land used in China. The cultivated land quality could be assessed via the following equation:

(1) $$CLQ=\sum_{i}^{n}w_{i}q_{i}$$

where *CLQ* represents the cultivated land quality, *n* is the number of selected indexes, *w_i_* is the weight of the index *i* for the cultivated land property and *q_i_* is the score of that index. The weights for quality indexes were determined by the analytic hierarchy process (AHP), and the consistency test (CR = 0.00872807 < 0.01) indicated a reasonable setting. The scoring criteria and the weight are presented in Table 1.

### 2.4. Land Productivity Estimation

To exhibit the spatial pattern of land productivity, we estimated the spatial distribution of grain yield by distributing the annual total grain yield in Dehui City and Jiutai District to 30 m × 30 m pixels according to the vegetation condition index (VCI) [18,30]. The grain yield statistics were derived from the yearbook of Changchun City. The estimation method could be described as Formulas (2) and (3):

(2) $${GY}_{ij}{=GY}_{j}{\times VCI}_{ij}/\sum_{i}^{n}{VCI}_{ij}$$

(3) $${VCI}_{ij}=\frac{{NDVI}_{i}-{NDVI}_{min}}{{NDVI}_{max}-{NDVI}_{min}}\times 100\%$$

where *GY_ij_* represents the annual grain yield of the i-th pixel in j-th county-level unit and *GY_j_* is the annual total grain yield in j-th county-level unit. Where *VCI_ij_* represents the VCI of the i-th pixel in j-th county-level unit, indicating a normalization process on the basis of NDVI, n is the number of pixels in the region; *NDVI_i_* is annual average NDVI value for the i-th pixel; and *NDVI_max_* and *NDVI_min_* represent the maximum and minimum annual average NDVI values across the region, respectively. The time-series NDVI data adopted were computed by using the Landsat OLI images (path/row: 118/029).

### 2.5. Ecosystem Services Evaluation

To reveal the changes in ESV before and after the reclamation of cultivated land reserves, we used 3 × 3 km grids that covered the reclaimed area. The ESV could be calculated via the following equation:

(4) $$ESV=\sum \left(A_{i}{\times VC}_{i}\right)$$

where *ESV* is the ESV in each grid, *A_i_* is the area of i-th land ecosystem (in ha) and *VC_i_* is the ESV per unit area of i-th land ecosystem.

Owing to the fact that the most cited research on evaluating ESV in China (that by Xie [31]) was performed on a national scale, it was inappropriate for directly calculating ESV in the study area. Thus, we further developed a per hectare ESV table (Table S2) based on the net primary productivity (NPP) correction along with previous research conducted in western Jilin Province [29]. Moreover, to specify the ESV of a farmland ecosystem that was not discussed in Li’s study, we also used the calculation results of per unit area ESV in different farmland ecosystems as conducted by Zhao [32]. The NPP correction could be described as follows:

(5) $${VC}_{i}{=VC}_{wi}\times \frac{{NPP}_{i}}{{NPP}_{wi}}$$

where *VC_i_* is the ESV per unit area of i-th land ecosystem; *VC_wi_* is the ESV per unit area of i-th land ecosystem in western Jilin Province; *NPP_i_* is the net primary productivity of i-th land ecosystem in the study area; and *NPP_wi_* is the net primary productivity of i-th land ecosystem in western Jilin Province.

## 3. Results and Analysis

### 3.1. Conversion of Cultivated Land Reserves during 1996–2015

**Figure 3:**
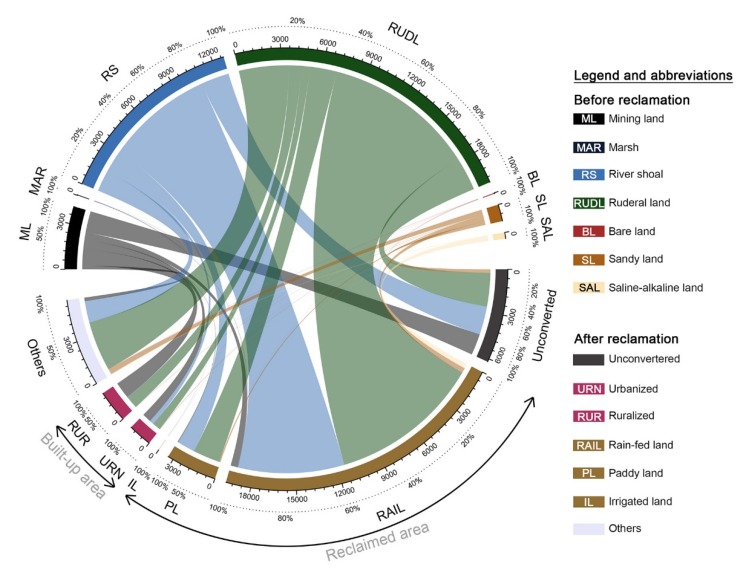
Chordal graph for the conversion of cultivated land reserves during 1996–2015.

From 1996 to 2015, an area of 32,500.55 ha cultivated land reserves was converted to other land resources, with only 16.02% of the original total remaining unconverted and nearly 60% being reclaimed as cultivated lands (Figure 3). In 2015, the proportion of reclaimed cultivated lands amounted to just 4.53% of the total. This indicates that the exploitation of cultivated land reserves in the study area has been prominent ever since the implementation of the requisition–compensation balance policy. The reclaimed cultivated lands, as always, were dominated by rain-fed lands, which accounted for over 85% of the total reclaimed area. Moreover, 4.03% of the total cultivated land reserves were being urbanized and 5.49% were being ruralized, respectively. Most of the urbanized or ruralized cultivated land reserves were mining land; this was evidence that the rural expansion against the population outflow in northeast China was also a considerable threat to natural resources when compared with urbanization.

Among all the cultivated land reserves, it was found that the amount of converted river shoal (10,753.15 ha) and ruderal land (17,780.72 ha) were the largest; indeed, over 90% of the reclaimed cultivated lands in 2015 were converted from river shoal and ruderal land. However, unlike rain-fed land and paddy land, nearly 60% of the irrigated lands were reclaimed from mining land-the other 40% were reclaimed from ruderal land. Marsh (49.01 ha) and bare land (48.89 ha) in the study area were found to be the least in quantity, accounting for only 0.27% and 0.18% of the total reclamation, respectively. The majority of sandy land and saline-alkaline land was also being reclaimed—87% of the saline-alkaline lands being converted to rain-fed land. Marsh, bare land and saline-alkaline land were found to be the most thoroughly converted of the cultivated land reserves.

**Figure 4:**
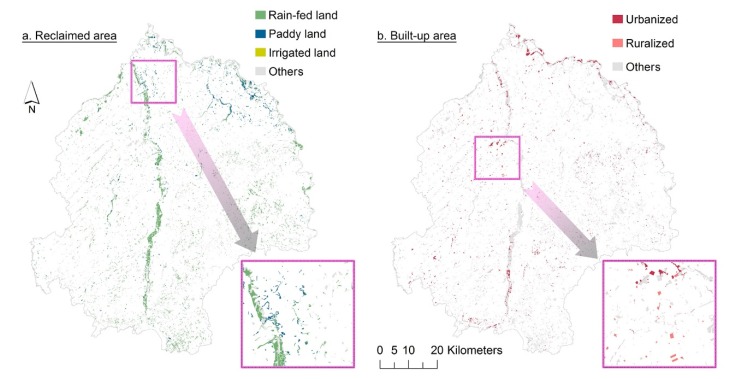
(**a**,**b**) Spatial distribution of reclaimed cultivated lands and built-up areas in the study area.

Spatially, most of the cultivated lands reclaimed from river shoals were distributed along the river bank of the Yinma River, a tributary of the Songhua River situated in the middle of the study area (Figure 4a). Meanwhile, in comparison with the distribution of cultivated land reserves, it can be seen that cultivated lands reclaimed from ruderal lands were smaller in scale, more dispersed and fragmented. The reclaimed paddy land, however, was mainly concentrated at the north-east side of the study area. Moreover, the urbanized cultivated land reserves were mainly clustered around the urban built-up area of Dehui City and Jiutai District (Figure 4b). However, the distribution of ruralized cultivated land reserves was mostly scattered, though the total amount was considerable.

### 3.2. Performance Evaluation of Reclaimed Cultivated Land Reserves

**Figure 5:**
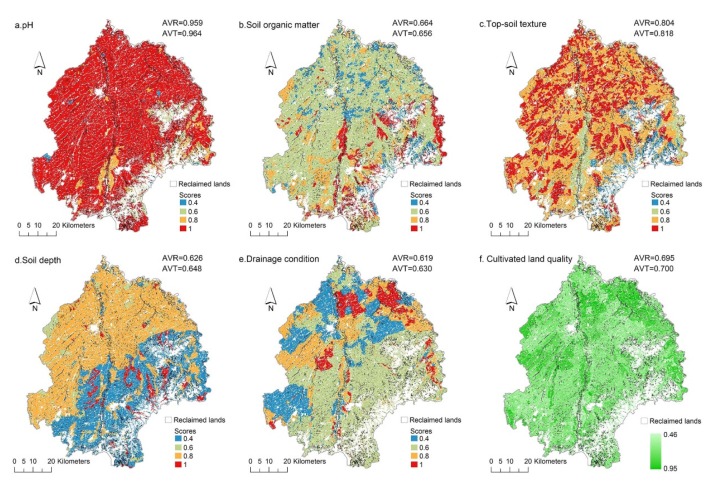
(**a**–**f**) Quality distribution and comparison between reclaimed cultivated lands and the total (AVR is the average value for reclaimed cultivated lands; AVT is the average value for cultivated lands in total).

First, the average size of cultivated lands reclaimed from river shoal and saline-alkaline land was 3.435 ha and 2.677 ha (Table 2), respectively. These results dwarfed the other figures, but they still could not be mentioned in the same breath as the population mean of 6.023 ha. This indicated that reclaimed cultivated lands were more fragmented compared with cultivated lands located in the traditional farming area. Additionally, and according to the quality assessment results, it was revealed that the average values for soil pH, top-soil texture, soil depth and drainage condition in the reclaimed area were slightly lower than the average for total cultivated lands, except for soil organic matter (Figure 5a–e). Furthermore, the average of total cultivated land quality exceeded the reclaimed cultivated lands (Figure 5f), which indicated that the reclaimed cultivated lands were inferior to the overall cultivated lands in quality, albeit they were very nearly the same.

The statistics showed that the cultivated lands reclaimed from bare land and sandy land scored higher in soil pH and soil depth, and cultivated lands reclaimed from marsh, river shoal and ruderal land were rich in soil organic matter (Table 2). Moreover, after proper soil amelioration, it could be found that the cultivated lands reclaimed from sandy land and saline-alkaline land showed an advantage in top-soil texture and drainage condition, and they were the best in the comprehensive quality rank. However, on the whole, the cultivated lands reclaimed from different land resources varied only slightly from one another.

**Figure 6:**
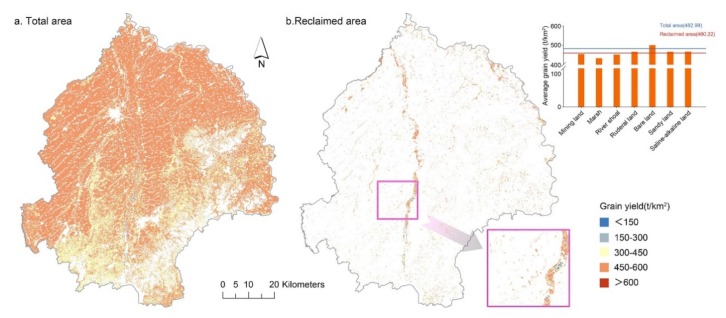
(**a**,**b**) Grain yield distribution and the difference between reclaimed cultivated lands in the study area.

The estimated grain yield distribution exhibited that, in general, cultivated lands located in Dehui City were more productive than the hilly Jiutai District (Figure 6a). More importantly, the average grain yield in the reclaimed area (460.22 t/km^2^) was evidently lower than the average of total cultivated lands (482.92 t/km^2^). This result indicated that-similar to the quality performance-the reclaimed cultivated lands were also inferior to the overall cultivated lands in productivity. The cultivated lands reclaimed from bare land (500.322 t/km^2^) exceeded the average of total cultivated lands in grain yield, but the reclaimed quantity was far too little. As for the rest of the reclaimed cultivated lands, they were all less productive, in particular for cultivated lands reclaimed from mining land, marsh and river shoal. A sketch of the river shoal reclaimed area showed that the grain output was mostly undesirable (Figure 6b).

### 3.3. Changes of Ecosystem Services in the Reclaimed Area

**Figure 7:**
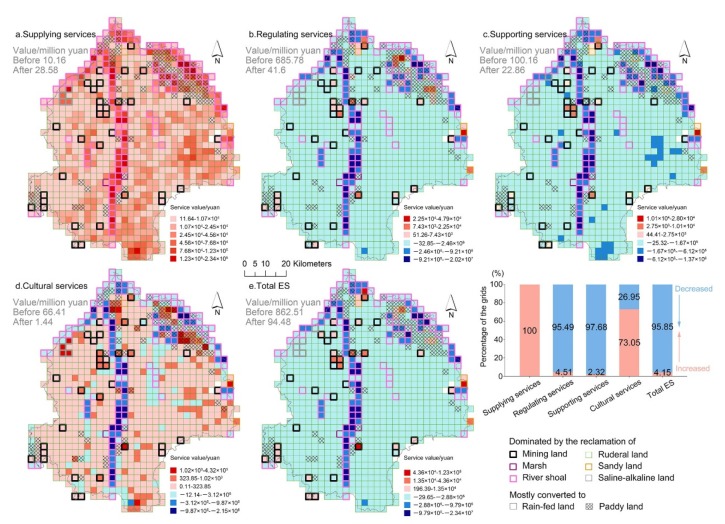
(**a**–**e**) Spatio-temporal variations of ecosystem service value in reclaimed area (the grid size is 3 km × 3 km; ES is the abbreviation of Ecosystem Services).

Before the cultivated land reserves were reclaimed, the ecosystem services in the reclaimed area were mainly composed of regulating services, and the values of supplying services were the lowest (Figure 7a,b). After the reserves were reclaimed as cultivated lands, the supplying service value increased by 18.42 million yuan, but there was a substantial decrease (768.03 million yuan) in the total ESV, and the decrease in regulating service value alone amounted to 644.18 million yuan (Figure 7a,b,e). Likewise, the values in supporting services and cultural services after the reclamation decreased by 77.3 and 66.97 million yuan, respectively (Figure 7c,d). Therefore, it can be concluded that the large-scale loss of reserves to cultivated lands had resulted in a sharp decline in ESV in the reclaimed area.

More specifically, the distribution of grids representing changes in ESV indicated that the supplying service values in the whole reclaimed area were all elevated, in particular on the north-east side where most of the paddy land was reclaimed, and along the riverbank where most of the river shoals were lost (Figure 7a). However, as for regulating services and supporting services, only a small part of the grids dominated by the reclamation of mining land showed an increase, the values of other grids were mostly decreased, especially in the grids reclaimed from river shoals (Figure 7b,c). The same distribution of grids with decreased values was also detected for cultural services (Figure 7d). Furthermore, even if the grids with elevated cultural service value accounted for 73.05%, the increase was considered to be relatively modest, mainly because the prevalent enhancing of supplying services and cultural services apparently could not offset the loss of total ecosystem services resulting from reclamation (Figure 7e). The variations of ESV indicated that the reclamation of mining land could improve the indigenous ecosystem services in all respects, but unfortunately the reclaimed area was relatively little. However, the reclamation of river shoal and ruderal land, i.e., the primary sources for cultivated land compensation, had resulted in the substantial and widely distributed decline in values of most ecosystem services.

## 4. Discussion

### 4.1. Gain and Loss via Reclamation of Cultivated Land Reserves in Northeast China

Under the impacts of the requisition–compensation balance policy, most of the cultivated land reserves in northeast China were reported to be reclaimed in order to compensate for losses in eastern coastal areas [8,33]. Taking the study area as an example, nearly 60% of the cultivated land reserves were reclaimed; over 20% of the land was occupied by other anthropogenic activities such urbanization from 1996 to 2015. The gain of 23,018.55 ha of reclaimed cultivated lands accounted for 4.53% of the total amount, contributing approximately 4.32% of the total grain output. The increase of cultivated lands in northeast China is indeed beneficial to maintaining its position in national grain production to a certain extent. However, there are also concerns that the overexploitation of cultivated land reserves is likely to incur the exhaustion of land resources for future reclamation [27,34], especially given that urbanization in China is expected to continue growing [35]. Moreover, previous studies have shown that the reclaimed cultivated lands are inferior to the lands being occupied by urbanization: thus, there possibly starts a threat to food security due to the consequent quality imbalance [16,36]. The present study further indicates that the reclaimed cultivated lands are of relatively poor quality and low productivity compared with cultivated lands as a whole rather than just the occupied lands. Results suggest that the reclaimed cultivated lands are serviceable, but may well not be adequate for functioning like cultivated lands elsewhere, apart from the reclaimed areas.

However, the results illustrated that this gain of cultivated lands in the study area was at the expense of 768.03 million yuan worth of ecosystem services. The aforementioned telecoupling analysis in well-developed areas of China has evidenced that the total ESV will be reduced as the requisition–compensation balance policy progresses, but the impacts of urbanization or agricultural restructuring were not isolated in that study [18]. Our results indicated that when specifically targeting the reclaimed area, the total ESV experienced a sharp decline, especially in regulating services and supporting services. The slight increase in supplying services and cultural services was far from sufficient for offsetting the loss in total ESV. More importantly, we argue that the promoted supplying services, including the production of food and raw materials, are a significant nationwide-even a worldwide-contribution considering that northeast China is one of the most crucial grain bases [28]. However, other ecosystem services in northeast China, which are indispensable for protecting the indigenous environment and for building sustainable agriculture, are mostly impaired because of the requisition–compensation balance policy. In a way, the policy may have sacrificed the indigenous ecosystem services in northeast China for ensuring national grain security.

**Figure 8:**
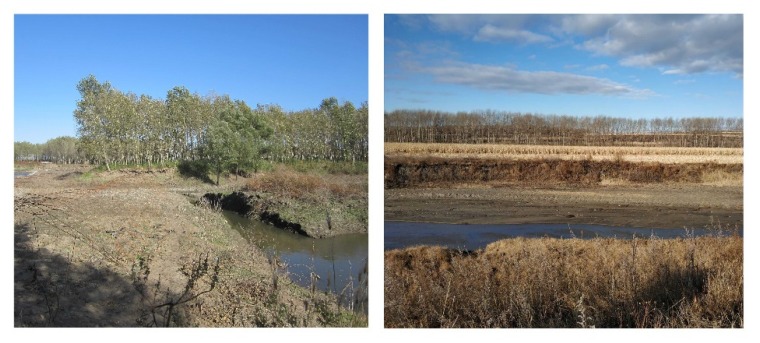
Cultivation in close proximity to a river in northeast China (sources: the authors).

It is worth noting that a large portion of reclaimed cultivated lands were converted from river shoal, and the field survey did confirm that there were many land parcels being cultivated adjacent to the river (see Figure 8). The naturally fertile soil and easier procedures of river shoal reclamation have made the river banks a focus of the reclaimed area. Unfortunately, besides the severe loss of ESV revealed in the present study, the reclamation of river shoal was also a great threat to the local water quality—cultivating crops next to the river enhances the chance of discharging pesticide residues and fertilizer directly into the water body [37,38]. Compared to farmland management in Germany or other European countries, cultivated lands in northeast China lack buffer-strips along river banks to isolate the water body from direct cultivation impacts [39,40]. On the other hand, the naturally fertile soil in northeast China is followed by an overload of instructions on how to manage the proper use of agricultural chemicals, leading to the prevalent single-dose and excessive applications [41], which undoubtedly exacerbates the pollution risk for local rivers. Furthermore, cultivated lands reclaimed from river shoal or sea-shore are considered as unstable, and they can hardly serve the same function as cultivated lands in traditional farming areas [34,42]. These unstable cultivated lands (including the river shoal–reclaimed lands) are to be thoroughly investigated in the third national land resources survey during 2017–2019, in an effort to get a clear picture of China’s cultivated land resources. Therefore, according to the gain and loss weighing, river shoal may be the most unworthy of the cultivated land reserves to be reclaimed.

### 4.2. Policy-Making Implications for Future Reclamation

The intrinsic distribution characteristics of land resources in China determine that the northeast region has to play a major part in shouldering the responsibility of compensating for the urbanization-induced cultivated land loss [8,33]. Consequently, the adverse effects brought on by agricultural expansion associated with the requisition–compensation balance policy seems inevitable, but yet mitigable. To begin with, and as mentioned above, the reclamation of river shoal should be restricted in consideration of their poor performance, the environmental risks and ESV loss. The cultivated lands reclaimed from sandy lands and saline-alkaline lands were superior to other reclaimed cultivated lands in quality and productivity, and they shared only a small proportion of the decline in total ESV. Moreover, the treatment of sandy land and saline-alkaline land and has been an important issue in regional environmental governance because desertification and salinization are counted as major causes of land degradation [43,44]. Thus, the reclamation of sandy land and saline-alkaline land should be prioritized. In addition, the cultivated lands reclaimed from mining lands were of poor quality and low grain yield, but the ecosystem services were mostly enhanced. Reclamation of mining land is a practical process involving land suitability assessment, top-soil stripping, ecological restoration, etc., which involves more time and effort [45,46]. Even so, reclamation is highly recommended since derelict mining land is considered a waste of land resource as well as a potential threat to the environment [46,47].

Grasslands and woodlands are commonly deemed as the primary sources for reclamation, and they are also the ecosystems being impaired globally by agricultural expansion [7,10]. A land use change study in China indicates that the conversion of grassland and woodland to cultivated lands has been prominent since the 1980s [8]. However, the reclamation of forest is gradually declining due to recognition of the significant role of forests in maintaining ecosystem services [48], and it was not included as one of the representative cultivated land reserves in the aforementioned national surveys. Ruderal land is a sub-category of grassland according to the national standard for classifying land use, and it is the most widely distributed and the largest cultivated land reserve in the study area. The results indicated that cultivated lands reclaimed from ruderal land were relatively productive, and the decline in total ESV was more moderate than the reclamation of river shoal or marsh. Given that the ruderal lands were mostly fragmented already, the reclamation of such cultivated land reserves was considered acceptable.

The basic intention of the requisition–compensation balance policy is to ensure national food security by maintaining the dynamic balance of cultivated lands [4,16], but it has resulted in the damage of natural ecosystems, especially in the north-east part of China. The ecological conservation in this region is, therefore, necessary for protecting vulnerable indigenous agroecosystems and also for programming grain production in a sustainable environment. Practical approaches for conserving ecosystem services in agriculture can be classified broadly as land-sparing and land-sharing strategies [49]. In terms of land-sparing strategies, there are certain methods (such as the delineation of protected areas that restricts irrational farming expansion in tropics [7,50], and the so-called Grain–for–Green strategy in China that reconverts slope cropland to woodland [26]) that could be imitated. For example, a protected area for cultivated land reserves with crucial ecosystem services should be demarcated. Moreover, land suitability assessment before reclamation projects needs to consider the potential loss in ESV, and an ecological baseline should be involved for basic quantity control.

Other than that, the land-sharing methods are considered more applicable for major reclaimed areas, as they are intended to preserve or to increase the ecological value of in-situ agroecosystems [21,49]. On one hand, it is suggested that the agricultural structure in northeast China is relatively simple and needs to implement proper restructuring to adapt to demand and be multi-functional [28]. More natural or semi-natural elements should be introduced to production-oriented cultivated land. In doing so, the within-field ecosystem services in both the traditional cultivated area and the reclaimed area can be increased to offset the loss of ESV following inevitable reclamation. On the other hand, the currently available farming practices in northeast China are thought to be unfriendly to agroecosystems, through, for example, the overuse of fertilizer [41], which can be a major threat to indigenous soils and water considering that there are reclaimed lands neighboring rivers [37,38]. Farming practices should, therefore, also be modified to improve the agricultural environment in reclaimed areas.

## 5. Conclusions

The trade-off in balancing food security and ecological security under conditions of rapid urbanization is a global problem. Ever since the implementation of the requisition-compensation balance policy of cultivated lands northeast China has been characterized by little occupation but massive compensation, losing numerous cultivated land reserves to agriculture. Previous studies have concentrated on investigating whether an overall balance has been achieved in well-developed regions, but few efforts have been made to weigh the indigenous gain and loss before and after reclamation in northeast China. We therefore selected two representative county-level units in northeast China-Dehui City and Jiutai District-as the study area. We evaluated the performance of cultivated lands reclaimed from different reserves in terms of quality and productivity, and further calculated the reclamation-induced ESV changes.

The results firstly indicated that nearly 60% of the cultivated land reserves were reclaimed during the period 1996–2015, the primary sources for cultivated lands compensation being river shoal and ruderal land. Marsh, bare land and saline-alkaline land were the most thoroughly converted cultivated land reserves. The reclaimed cultivated lands were found to be more fragmented, and they were also inferior to cultivated lands as a whole in quality (although they were very nearly the same). Moreover, the reclaimed lands were less productive, in particular mining land, marsh and river shoal-reclaimed cultivated lands. Besides the gain in the amount of cultivated lands, the reclamation also resulted in a substantial decrease (768.03 million yuan) in the total ESV. The slight promotion of supplying services and cultural services could not offset the severe loss in regulating services and supporting services. This conclusion implies that the requisition–compensation balance policy was beneficial for maintaining the position of northeast China in the national food security system, but it was highly likely that the loss of cultivated land reserves would hamper the delivery of local ecosystem services, and be detrimental to the development of sustainable agriculture.

In order to ease the adverse ecological consequences resulting from improper reclamation, we suggested that some reclamation, e.g., river shoal and marsh, should be deprecated and avoided if at all possible. In contrast, the reclamation of mining lands and unutilized lands including sandy land and saline-alkaline land, should be prioritized. Furthermore, the land-sparing scheme was considered necessary for improving the agricultural environment in northeast China because agricultural restructuring and modified farming practices were already recommended. Only by protecting indigenous ecosystems can agricultural production in major reclaimed areas be long-lasting and sustainable.

## Figures and Tables

**Table 1:** Assessment system for cultivated land quality in the study area.

Quality Index	Scores				Weights
	1	0.8	0.6	0.4	
pH	6.0–7.8	5.5–5.9 or 7.9–8.4	5.0–5.4 or 8.5–8.9	others	0.08
Soil organic matter	≥4%	3–4%	2–3%	<2%	0.29
Top-soil texture	Loamy soil	Clay soil	Sandy soil	Gravel soil	0.21
Soil depth	≥90cm	80–89cm	70–79cm	<69cm	0.09
Drainage condition	Complete	Basically complete	Poor	No drainage	0.33

**Table 2:** Comparison of quality indexes between cultivated lands reclaimed from different reserves.

Reclaimed From	Average for Quality Index						
	Size	pH	Soil Organic Matter	Top-Soil Texture	Soil Depth	Drainage Condition	Comprehensive Quality
Mining land	0.253	0.968	0.638	0.812	0.661	0.616	0.690
Marsh	0.701	0.972	0.688	0.824	0.528	0.624	0.702
River shoal	3.435	0.988	0.666	0.815	0.696	0.636	0.711
Ruderal land	0.572	0.954	0.669	0.799	0.611	0.617	0.693
Bare land	0.215	0.990	0.588	0.895	0.787	0.523	0.672
Sandy land	0.808	0.990	0.622	0.876	0.732	0.696	0.729
Saline-alkaline land	2.677	0.985	0.597	0.946	0.784	0.657	0.724

## Supplementary Materials

The following are available online at https://www.mdpi.com/1660-4601/17/4/1211/s1, Table S1: Land use classification and detailed connotations in the study, Table S2: Ecosystem service value (ESV) per hectare of different terrestrial ecosystems in the study area.
