# Supplementary material for: Reclamation of Cultivated Land Reserves in Northeast China: Indigenous Ecological Insecurity Underlying National Food Security
Source: Int J Environ Res Public Health. 2020 Feb 13;17(4):1211. doi: 10.3390/ijerph17041211 (PMC7068416; doi:10.3390/ijerph17041211)
Supplement: Supplementary file 1 [file ijerph-17-01211-s001.pdf]

Table S1 Land use classification and detailed connotations in the study.

Land use category	Connotations
Rain-fed land	Rain-fed land refers to cultivated lands that have no irrigation facilities and are utilized for the cultivation of xerophyte.
Paddy land	Paddy land refers to cultivated lands that are used for the cultivation of rice.
Irrigated land	Irrigated land refers to cultivated lands that are provided with irrigation water and facilities.
Mining land	Mining land refers to lands that are utilized for mining, quarrying or sand excavation.
Marsh	Marsh refers to lands that are perennially waterlogged and grow hygrophyte.
River shoal	River shoal refers to the intertidal area by river or lake.
Ruderal land	Ruderal land refers to natural grasslands that are not utilized for grazing.
Bare land	Bare land refers to bare soil without vegetation cover.
Sandy land	Sandy land refers lands that are covered by sand.
Saline-alkaline land	Saline-alkaline refers lands that are salinized and grow salt-tolerant plants.

Table S2 Ecosystem service value (ESV) per hectare of different terrestrial ecosystems in the study area (yuan/ha/yr).

		Reclaimed from						Reclaimed as		
Land use		Marsh	River shoal	Ruderal land	Bare land	Sandy land	Saline-alkaline land	Rain-fed land	Paddy land	Irrigated land
**Ecosystems**		**Wetland**	**Wetland**	**Grassland**	**Desert**	**Desert**	**Desert**	**Farmland**	**Farmland**	**Farmland**
Supplying services	Food production	385.76	397.73	383.88	8.8	8.8	179.69	826.02	1303.91	1154.87
	Raw material	89.94	92.73	63.93	0	0	29.85	388.71	86.29	54.56
	Sub-total	475.7	490.46	447.81	8.8	8.8	209.54	1214.73	1390.2	1209.43
Regulating services	Gas regulation	2314.15	2385.93	1023.64	0	0	479.24	651.1	1064.22	809.32
	Climate regulation	21984.75	22666.73	1151.51	0	0	539.08	349.84	546.49	427.39
	Water conservation	19927.78	20545.95	1023.64	26.5	26.5	479.24	262.38	2607.82	1727.76
	Waste disposal	23373.36	24098.41	1676.15	8.8	8.8	784.62	97.18	162.99	127.31
	Sub-total	67600.04	69697.02	4874.94	35.3	35.3	2282.18	1360.5	4381.52	3091.78
Supporting services	Soil formation & retention	2198.49	2266.69	2494.95	17.7	17.7	1168.01	1000.94	9.59	9.09
	Biodiversity protection	3214.26	3313.97	1394.62	300.8	300.8	652.93	126.33	201.34	154.59
	Sub-total	5412.75	5580.66	3889.57	318.5	318.5	1820.94	1127.27	210.93	163.68
Cultural services	Recreation & culture	7135.39	7356.74	51.17	8.8	8.8	24	58.31	86.29	72.75
	Total ESV	80623.88	83124.88	9263.49	371.4	371.4	4336.66	3760.81	6068.94	4537.64

Note: 100 yuan=16.06 dollars in 2015
